# Prevalence of Ventilator-Associated Pneumonia in Children Admitted to Pediatric ‎Intensive Care Units in the Middle East: A Systematic Review

**DOI:** 10.7759/cureus.51230

**Published:** 2023-12-28

**Authors:** Hassan T Mohamed, Wail Abdullah Farhan Alenezi, Muhannad Abdullah A Alanzi, Faris Ibrahim Saleh Alsuqub, Salem Ali Salem Alhazmi, Omar Madhi Mohammed Alhazmi

**Affiliations:** 1 Pediatrics, Maternity and Children Hospital, Arar, SAU; 2 Pediatric Medicine, Northern Border University, Arar, SAU; 3 Medicine, Northern Border University, Arar, SAU

**Keywords:** pediatric pneumonia, pneumonia, pediatric, nosocomial infections, ventilator-associated pneumonia

## Abstract

Ventilator-associated pneumonia (VAP) is a common healthcare-associated disease in intensive care units, leading to significant morbidity and mortality. This systematic review aims to investigate the prevalence, risk factors, and prevention strategies for VAP in the Middle East. PubMed, SCOPUS, Web of Science, Science Direct, and Google Scholar were systematically searched to include the relevant literature. Rayyan QCRI was used throughout this systematic approach. Ten studies, involving a total of 6295 patients diagnosed with VAP, were included in this review. Among these patients, 336 (5.3%) developed VAP. The prevalence of VAP in children and neonates in the Middle East was relatively low. Risk factors associated with VAP development included prematurity, low birth weight, prolonged mechanical ventilation, enteral feeding, intrusive devices such as umbilical catheters, and cardiac operations. All reviewed studies emphasized the importance of infection control measures in reducing the risk of VAP.

## Introduction and background

Pneumonia is the most prevalent infection in children requiring hospitalization and the main reason for antibiotic prescriptions in pediatric hospitals. Ventilator-associated pneumonia (VAP) and community-acquired pneumonia (CAP) are two types of pediatric pneumonia [1]. Investigating the different viruses that result in PICU admissions could be useful in organizing healthcare resources and comprehending the local epidemiology. During seasonal crises or infectious disease outbreaks, like the COVID-19 pandemic, this is especially crucial. Both patients and healthcare officials were concerned about overcrowding medical facilities as the virus spread [2].

In the world today, CAP is one of the top causes of death in children, especially those under the age of five [3,4]. Despite decreased death rates in industrialized countries, CAP remains the most common cause of pediatric hospitalization outside of the neonatal period in the United States [1].

Additionally, between 12% and 20% of pediatric CAP patients require critical care [1,5,6], frequently because of pneumonia and septicemia [3], as well as the development of respiratory failure requiring assisted ventilation. Severe CAP can occur as a result of underlying comorbidities such as preterm birth, bronchopulmonary dysplasia, congenital heart disease, immunodeficiency, and severe cerebral palsy. Another risk factor is having a relevant medical history of severe pneumonia [3,7].

Bloodstream infections are the second most common hospital-acquired illness (HAI) in children, after VAP. In pediatric intensive care units (PICUs), patients on mechanical ventilation (MV) are at risk of developing VAP, which is believed to occur in 10-20% of ventilated patients. Similar to CAP, VAP entails a significant risk of morbidity and mortality; it can lengthen the time spent in the hospital and receiving respiratory assistance, increase mortality rates, and have a negative influence on patient outcomes in PICUs [6,8,9]. Under one year of age, a compromised immune system, unanticipated emergency intubations and reintubations, acute respiratory distress syndrome, continuous enteral feeding, and sedation interruption have all been associated with an increased risk of developing VAP [8-10].

VAP and severe CAP have diverse etiologies, pathophysiologies, and microbiological outcomes that call for PICU hospitalization. It is essential to recognize the most common bacterial infections in order to aid in decisions made with empirical antibiotics [11].

According to a recent Saudi study, respiratory virus infections account for a sizable number of PICU admission days where MV is required. Multiple viral infection patients had greater comorbidities, longer PICU length of stay, and higher median pediatric index of mortality (PIM) 2 scores [12]. 

The bacterial pathogens most often associated with CAP are known to be gram-positive and non-Enterobacterales gram-negative bacteria, such as *Streptococcus*​​​​ *pneumoniae*, *Staphylococcus aureus*, *Haemophilus*​​​​​​​* influenzae*, and *Moraxella catarrhalis* [13]. In contrast, the most frequently identified bacterial pathogens in VAP are Enterobacterales and other gram-negative bacteria [9]. Patients with VAP are more likely to have germs that are widely and multiple-drug resistant, according to the susceptibility pattern [14]. This makes controlling VAP in an ICU challenging. Understanding the effects of VAP in any context is essential for improving and carrying out preventative actions. The prevalence, risk factors, and prevention of VAP in the Middle East were examined in this systematic review.

## Review

Methodology

The PRISMA (Preferred Reporting Items for Systematic Reviews and Meta-Analyses) guidelines were followed in conducting this systematic review [15].

Study design and duration

This systematic review was carried out in August 2023.

Search strategy

To locate the pertinent literature, a thorough search was conducted across five major databases, including PubMed, SCOPUS, Web of Science, Science Direct, and Google Scholar. We limited our search to English and took into account the particular needs of each database. To discover the pertinent papers, the following keywords were translated into PubMed Mesh terms: "Ventilator-associated pneumonia," "nosocomial infections," "Children," "Paediatric," "Middle East," "Arab countries," and "Prevalence." The essential keywords were matched using the Boolean operators "OR" and "AND". The search returned a list of publications containing complete English text, free papers, and human trials.

Table 1 shows that the inclusion and exclusion criteria for the study on the prevalence, risk factors, and prevention of VAP in the Middle East are clearly outlined. By specifying the study designs that are eligible for inclusion, such as clinical studies, and excluding non-English language publications, case reports, review articles, systematic reviews, and meta-analyses, the researchers are ensuring that the data included in their study is of high quality and relevant to their research question.

**Table 1:** Selection criteria of the included studies.

Inclusion criteria	Exclusion criteria
Study designs that looked at the prevalence, risk factors, and prevention of VAP in the Middle East	Non-English language
Clinical studies	Case reports, review articles, systematic review, and meta-analysis
Children (birth to 18 years old)	Adults (18 years or older)

Data extraction

Rayyan (QCRI) was utilized to look for duplicate information in the search strategy's output [16]. The researchers changed the combined search results using a set of inclusion/exclusion criteria to assess the relevancy of the titles and abstracts. The reviewers extensively reviewed each manuscript that met the conditions for inclusion. The authors addressed dispute resolution techniques. The authorized research was submitted utilizing a data extraction form that had already been created. The writers gathered information on the research titles, authors, study year, country, participants, gender, prevalence, and primary outcomes. A second sheet was prepared to analyze the possibility of bias.

Strategy for data synthesis

Summary tables were created using data from relevant studies to provide a qualitative interpretation of the findings and study components. After retrieving the data for the systematic review, the most efficient strategy to use the data from the included study articles was chosen.

Risk-of-bias assessment

The quality of the included studies was assessed using the ROBINS-I (Risk Of Bias In Non-randomised Studies - of Interventions) risk-of-bias assessment approach for non-randomized trials of treatments [17]. Confounding, participant selection for the study, classification of treatments, deviations from intended interventions, missing data, evaluation of outcomes, and selection of the reported result were among the seven themes examined.

Results

Search Results

**Figure 1:**
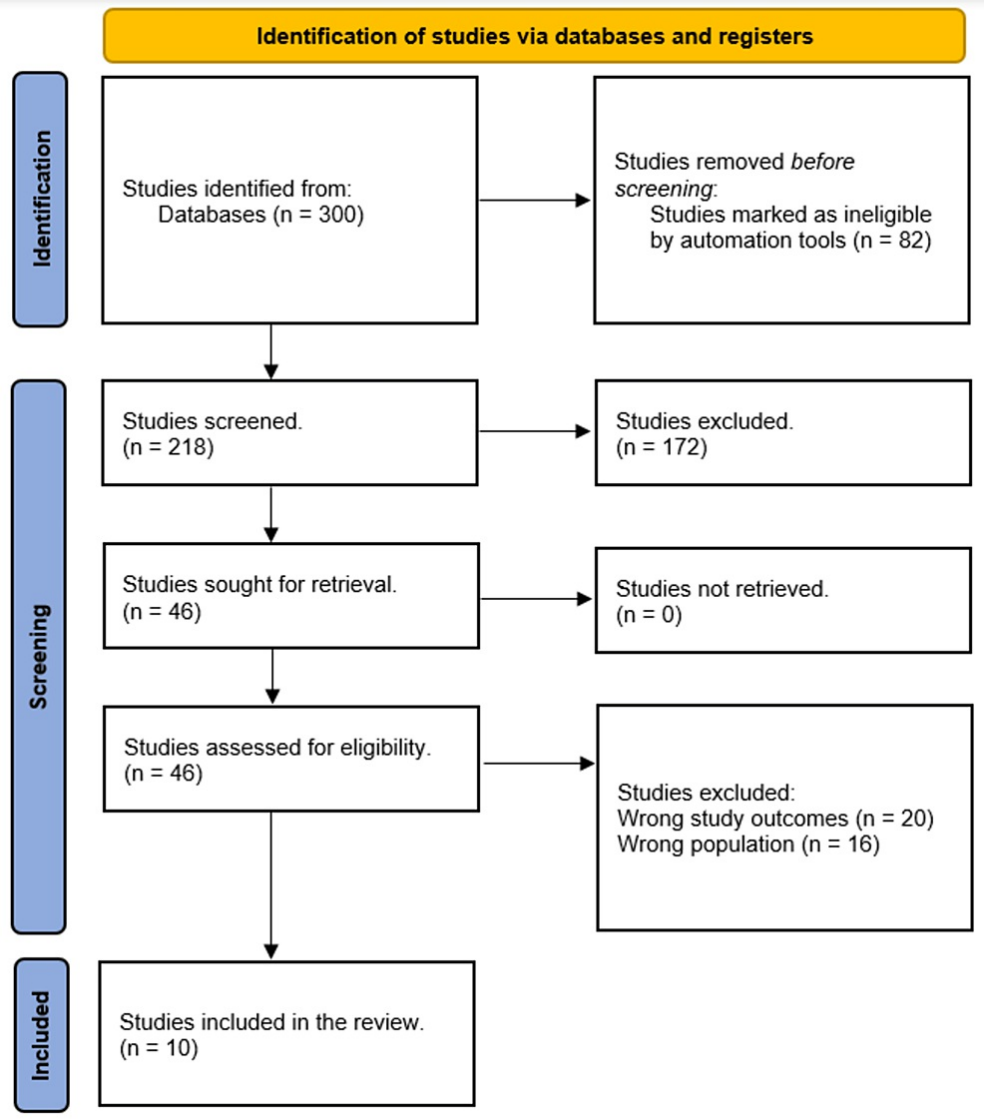
PRISMA flowchart summarizes the study selection process. PRISMA, Preferred Reporting Items for Systematic Reviews and Meta-Analyses.

The systematic search yielded 300 study papers, of which 82 duplicates were removed. Two hundred and eighteen papers were subjected to title and abstract screening, with 172 studies being rejected. There were 46 reports sought for retrieval, but no articles were found. Finally, 36 papers were screened for full-text evaluation; 20 were rejected due to incorrect research results, and 16 were rejected due to the incorrect population type. This systematic review comprised 10 research publications. Figure 1 depicts an overview of the study selection procedure.

Characteristics of the Included Studies

The sociodemographic features of the included study papers are presented in Table 2 [18-27]. Our findings comprised nine trials involving 6295 children with VAP. Sic investigations were carried out in Egypt [22-27], Saudi Arabia [18-20], and Kuwait [21]. Nine of the included studies [18-21,23-27] were prospective in nature, with just one being a randomized control trial [22].

**Table 2:** The sociodemographic characteristics of the included studies. NM, not mentioned; RCT, randomized controlled trials.

Study	Study design	Country	Participants	Mean age (months)	Males, n (%)
Osman et al. 2020 [18]	Prospective cohort	Saudi Arabia	141	12	69 (48.9)
Almuneef et al. 2004 [19]	Prospective surveillance	Saudi Arabia	361	28.6 ± 40.5	230 (63.7)
Shaath et al. 2014 [20]	Prospective surveillance	Saudi Arabia	137	19.7 ± 2.5	72 (52)
Al-Mousa et al. 2016 [21]	Prospective cohort	Kuwait	4403	NM	NM
El-Ella et al. 2022 [22]	RCT	Egypt	72	24	34 (47.2)
Rasslan et al. 2012 [23]	Prospective cohort	Egypt	473	NM	NM
El-Nawawy et al. 2006 [24]	Prospective observational	Egypt	216	15.9 ± 27.6	124 (57.4)
Meligy et al. 2017 [25]	Prospective observational	Egypt	293	8.5	162 (55.3)
Badr et al. 2011 [26]	Prospective observational	Egypt	56	33.6 ± 3.2	29 (51.7)
Azab et al. 2015 [27]	Prospective observational	Egypt	143	31.73 ± 4.3	95 (66.4)

Table 3 displays the clinical features. Three hundred and thirty-six (5.3%) of the total patients developed VAP. The primary risk factors for developing VAP include prematurity, low birth weight, prolonged artificial breathing, enteral feeding, invasive devices such as umbilical catheters, and cardiac operations [20,26]. One study found that *Candida albicans* infection was common in VAP patients [24]. Omeprazole and pre- and post-bundles were ineffective in preventing VAP [18,22]. All of the included studies revealed that infection control techniques are critical for preventing the spread of VAP. 

**Table 3:** Clinical characteristics and outcomes of the included studies. NA, not applicable; VAP, ventilator-associated pneumonia; NNIS, National Nosocomial Infections Surveillance; CPB, cardiopulmonary bypass; TPN, total parenteral nutrition; PICU, pediatric intensive care unit; MODS, multiple organ dysfunction syndrome; PS, pressure support; SIMV, synchronized intermittent mandatory ventilation; NB-BAL, non-bronchoscopic bronchoalveolar lavage; ROBINS-I, Risk Of Bias In Non-randomised Studies - of Interventions.

Study	Prevalence of VAP, n (%)	Main outcomes	ROBINS-I
Osman et al. 2020 [18]	44 (31.2)	The use of a pre- and post-bundle had no effect on the incidence of VAP.	Moderate
Almuneef et al. 2004 [19]	37 (10.2)	The mean VAP rate at this hospital was higher than what the (NNIS) system, which monitors PICUs, reported. This work has established a benchmark for future research on VAP in Saudi Arabia's pediatric critical care population. More study in the field is needed to compare and develop preventative methods.	Moderate
Shaath et al. 2014 [20]	9 (6.6)	The prevalence of VAP in children after cardiac surgery is significantly greater than predicted in the PICU, particularly in those patients who require protracted cardiopulmonary bypass (CPB), total parenteral nutrition (TPN), or PCICU stays. A gram-negative bacteria is frequently the cause of VAP. VAP has a major influence on pediatric morbidity and mortality following heart surgery.	Moderate
Al-Mousa et al. 2016 [21]	17 (0.4)	In the PICU, the frequency of VAP in children following cardiac surgery is substantially higher than expected, particularly in those patients who require prolonged cardiopulmonary bypass (CPB), total parenteral nutrition (TPN), or PCICU stays. VAP is commonly caused by gram-negative bacteria. VAP has a significant impact on child morbidity and mortality after cardiac surgery.	Moderate
El-Ella et al. 2022 [22]	7 (9.6)	Omeprazole with SUP did not halt upper gastrointestinal bleeding in children with mild to severe organ failure at the time of PICU admission.	NA
Rasslan et al. 2012 [23]	35 (7.34)	Omeprazole with SUP did not halt upper gastrointestinal bleeding in children with mild to severe organ failure at the time of PICU admission.	High
El-Nawawy et al. 2006 [24]	34 (16)	The high NI rate (23%) was mostly attributable to the severity of the ailment at the time of admission, as seen by the high PRISM III score value.	Moderate
Meligy et al. 2017 [25]	80 (27.3)	Mechanical ventilation is commonly employed in PICUs. The vast majority of pediatric ventilators are used for neurological reasons. The most common ventilation approach is pressure control with SIMV and PS, whereas PS with CPAP is the ideal weaning strategy. A higher PRISM III score and MODS were related to a higher death rate.	Moderate
Badr et al. 2011 [26]	32 (57.1)	In our unit, the primary risk factors for developing VAP include prematurity, low birth weight, prolonged artificial breathing, enteral feeding, and invasive devices such as umbilical catheters. The vast majority of cultures obtained by NB-BAL are gram-negative bacteria.	Moderate
Azab et al. 2015 [27]	73 (51)	A set of infection control methods can greatly reduce the incidence of VAP during newborn breathing. This "VAP prevention bundle" might be applied to other NICUs in Egypt and other developing countries.	Moderate

Discussion

Understanding the etiology of VAP is crucial for creating treatment-guiding principles and prevention interventions [1]. Bacteria from the upper respiratory tract can aspirate into the trachea or lung, causing VAP when they reach the stomach or voice cords. A vast range of host defense mechanisms protect the trachea and lungs from bacterial infection [9]. However, as a result of malnutrition, chronic diseases, or immunosuppression, host defenses may be weakened in critically sick patients, particularly those on mechanical breathing. One of the most important aspects of this problem is the prevention of nosocomial infections. It is critical to identify risk factors and predictors in order to implement appropriate VAP measures.

The frequency of VAP in critically ill children in the PICU varies greatly depending on the environment and location [3]. In the Middle East, we found a 5.3% incidence among children and newborns on ventilator support. There has been an upsurge in studies in recent years emphasizing the need of using a number of key evidence-based methods to battle this avoidable HAI [4]. The lack of gold standards for VAP diagnosis emphasizes the importance of such precautions. The appropriate components for these preventative measures must be further investigated, especially for cost-benefit considerations and to determine their efficacy [26]. Many recent studies, however, have demonstrated that deploying VAP preventive bundles may result in lower VAP incidence and the necessity of such as a daily aspect of ICU patient care. VAP incidence varies in general, but it is usually excessively high. Differences in patient profiles, dietary circumstances, resource accessibility, and diagnostic standards, among other factors, might create variations. According to global pediatric research, VAP affects between 2% and 35% of mechanically ventilated children in the PICU [14].

The primary risk factors for developing VAP include prematurity, low birth weight, prolonged artificial ventilation, enteral feeding, invasive devices such as umbilical catheters, and cardiac operations [20,26]. All of the included studies revealed that infection control techniques are critical for preventing the occurrence of VAP. The majority of risk factors for developing VAP are well understood, with enteral feeding and the use of certain drugs, such as proton pump inhibitors or histamine-2 receptor blockers, being the most significant [28]. The former typically causes regurgitation, while the latter, by decreasing acid production, allows nosocomial infections to colonize the aerodigestive tract and endotracheal tube. Maintaining tracheal cuff pressure, reducing needless tracheal suction, avoiding excessive stomach distention, and avoiding equipment infection are also important considerations [12]. Furthermore, individuals with genetic diseases, neurological or cardiovascular illness, hospitalizations, or extended artificial breathing are at a greater risk of aspiration [3,17].

Gulf Cooperation Council - Centre for Infection Control (GCC-CIC) is in charge of infection control and surveillance throughout the GCC member countries. The GCC-CIC provides educational and training programs for staff members involved in infection prevention and control, as well as evidence-based guidelines for infection control practices [6]. The GCC Infection Prevention and Control Manual has thorough instructions for setting up and maintaining MV circuits and artificial airways, collecting specimens, using personal protective equipment, and practicing hand hygiene [22]. It also includes the most recent criteria for respiratory operations. There is currently no full and integrated system in place for obtaining and synthesizing data from hospitals across the area, and further initiatives and more broad involvement in infection surveillance and data management training programs are necessary [9].

Omeprazole and pre- and post-bundles were ineffective in preventing VAP [18,22]. Because there is a scarcity of data on the pediatric population, the identified risk factors are mostly drawn from academic research that examines this subject from the perspective of adults. The preventative techniques may be divided into two categories that are easier to understand: first, sophisticated preventive care, which involves the patient being cared for by the medical team. This would necessitate persistent hygiene, notably in terms of oral and hand hygiene, to which medical workers must closely adhere. The patient's head posture, proper feeding strategy, and medication administration are all included in this category. All of these are largely aimed at preventing ambition. Correct methods for artificial ventilation, suctioning techniques, changing ventilation circuits, and general equipment care and management would be included in the second group [29].

A common issue in bundle prevention is the provision of initiatives for adult patients but not for pediatric patients. De Cristofano et al.'s 2016 study showed that preventive bundles can be effective. They focused on adopting a VAP prevention bundle with the goal of improving the PICU's level of care and decreasing the VAP rate by 25% every six months during a two-year period. According to the findings of this study, the prophylactic bundle should include four critical components: an elevated head of bed, chlorhexidine-infused mouthwash, a clean, dry ventilator circuit, and daily withdrawal of IV sedatives. When the bundle was used, the mean VAP rate fell from 6.34 occurrences per 1000 ventilator days before the intervention to 2.38 [17].

The lack of gold standards for VAP diagnosis highlights the importance of such preventive actions. Even though many recent studies have shown that implementing ventilator prevention bundles reduces VAP rates and is important as part of the routine care of an ICU patient [5], the right components that should be included in these prevention strategies need to be further evaluated, primarily for cost-benefit considerations and efficacy.

The study provides valuable insights into the prevalence of VAP in PICUs in the Middle East. However, it is important to note the limitations of this study. One potential limitation is the potential for publication bias, as only published studies were included in the review. Additionally, the quality of the included studies and the heterogeneity of the data may also impact the reliability of the findings. It is important for readers to consider these limitations when interpreting the results of the study.

The findings of this systematic review can have significant future implications for healthcare providers, policymakers, and researchers. By understanding the prevalence of VAP in this specific population, healthcare professionals can develop targeted interventions and guidelines to reduce the incidence of this serious infection. Additionally, this study can also highlight the need for further research and investment in pediatric critical care facilities in the Middle East. Overall, this study has the potential to drive improvements in pediatric intensive care and ultimately improve the outcomes for children in the region.

## Conclusions

In the Middle East, the overall frequency of VAP among children and newborns was low. Prematurity, low birth weight, extended artificial breathing, enteral feeding, invasive devices such as umbilical catheters, and cardiac procedures are the primary risk factors for developing VAP. All of the research evaluated demonstrated the importance of infection control methods in lowering the risk of VAP. The research should also include information regarding care regimens, toolkits used to do root-cause analysis on VAP infections, and bundles used to minimize VAP incidence. By taking extra care of these risk factors, the incidence of VAP should be minimized. Meanwhile, more research on risk factors and identifying VAP in the PICU may be necessary.

## References

[1] Jain S, Williams DJ, Arnold SR (2015). Community-acquired pneumonia requiring hospitalization among U.S. children. N Engl J Med.

[2] Kang L, Jing W, Liu J, Liu M (2023). Trends of global and regional aetiologies, risk factors and mortality of lower respiratory infections from 1990 to 2019: An analysis for the Global Burden of Disease Study 2019. Respirology.

[3] McIntosh K (2002). Community-acquired pneumonia in children. N Engl J Med.

[4] Rudan I, O'Brien KL, Nair H (2013). Epidemiology and etiology of childhood pneumonia in 2010: Estimates of incidence, severe morbidity, mortality, underlying risk factors and causative pathogens for 192 countries. J Glob Health.

[5] Myers AL, Hall M, Williams DJ (2013). Prevalence of bacteremia in hospitalized pediatric patients with community-acquired pneumonia. Pediatr Infect Dis J.

[6] Dassner AM, Nicolau DP, Girotto JE (2017). Management of pneumonia in the pediatric critical care unit: An area for antimicrobial stewardship. Curr Pediatr Rev.

[7] Shan W, Shi T, Chen K (2019). Risk factors for severe community-acquired pneumonia among children hospitalized with CAP younger than 5 years of age. Pediatr Infect Dis J.

[8] Amanati A, Karimi A, Fahimzad A, Shamshiri AR, Fallah F, Mahdavi A, Talebian M (2017). Incidence of ventilator-associated pneumonia in critically ill children undergoing mechanical ventilation in pediatric intensive care unit. Children (Basel).

[9] Hamid MH, Malik MA, Masood J, Zia A, Ahmad TM (2012). Ventilator-associated pneumonia in children. J Coll Physicians Surg Pak.

[10] Chomton M, Brossier D, Sauthier M, Vallières E, Dubois J, Emeriaud G, Jouvet P (2018). Ventilator-associated pneumonia and events in pediatric intensive care: A single center study. Pediatr Crit Care Med.

[11] Harris M, Clark J, Coote N, Fletcher P, Harnden A, McKean M, Thomson A (2011). British Thoracic Society guidelines for the management of community acquired pneumonia in children: Update 2011. Thorax.

[12] Al-Eyadhy A, Almazyad M, Hasan G (2023). The burden of viral infections in pediatric intensive care unit between endemic and pandemic coronavirus infections: A tertiary care center experience. J Infect Chemother.

[13] Zhang Q, Guo Z, Bai Z, MacDonald NE (2013). A 4 year prospective study to determine risk factors for severe community acquired pneumonia in children in southern China. Pediatr Pulmonol.

[14] El-Nawawy A, Ramadan MA, Antonios MA, Arafa SA, Hamza E (2019). Bacteriologic profile and susceptibility pattern of mechanically ventilated paediatric patients with pneumonia. J Glob Antimicrob Resist.

[15] Page MJ, McKenzie JE, Bossuyt PM (2021). The PRISMA 2020 statement: an updated guideline for reporting systematic reviews. BMJ.

[16] Ouzzani M, Hammady H, Fedorowicz Z, Elmagarmid A (2016). Rayyan-a web and mobile app for systematic reviews. Syst Rev.

[17] De Cristofano A, Peuchot V, Canepari A, Franco V, Perez A, Eulmesekian P (2016). Implementation of a ventilator-associated pneumonia prevention bundle in a single PICU. Pediatr Crit Care Med.

[18] Osman S, Al Talhi YM, AlDabbagh M, Baksh M, Osman M, Azzam M (2020). The incidence of ventilator-associated pneumonia (VAP) in a tertiary-care center: Comparison between pre- and post-VAP prevention bundle. J Infect Public Health.

[19] Almuneef M, Memish ZA, Balkhy HH, Alalem H, Abutaleb A (2004). Ventilator-associated pneumonia in a pediatric intensive care unit in Saudi Arabia: A 30-month prospective surveillance. Infect Control Hosp Epidemiol.

[20] Shaath GA, Jijeh A, Faruqui F, Bullard L, Mehmood A, Kabbani MS (2014). Ventilator-associated pneumonia in children after cardiac surgery. Pediatr Cardiol.

[21] Al-Mousa HH, Omar AA, Rosenthal VD (2016). Device-associated infection rates, bacterial resistance, length of stay, and mortality in Kuwait: International Nosocomial Infection Consortium findings. Am J Infect Control.

[22] Abu El-Ella SS, El-Mekkawy MS, Mohamed Selim A (2022). Stress ulcer prophylaxis for critically ill children: Routine use needs to be re-examined. An Pediatr (Engl Ed).

[23] Rasslan O, Seliem ZS, Ghazi IA (2012). Device-associated infection rates in adult and pediatric intensive care units of hospitals in Egypt. International Nosocomial Infection Control Consortium (INICC) findings. J Infect Public Health.

[24] El-Nawawy AA, Abd El-Fattah MM, Metwally HA, Barakat SS, Hassan IA (2006). One year study of bacterial and fungal nosocomial infections among patients in pediatric intensive care unit (PICU) in Alexandria. J Trop Pediatr.

[25] Meligy BS, Kamal S, El Sherbini SA (2017). Mechanical ventilation practice in Egyptian pediatric intensive care units. Electron Physician.

[26] Badr MA, Ali YF, Albanna EA, Beshir MR, Amr GE (2011). Ventilator associated pneumonia in critically-ill neonates admitted to neonatal intensive care unit, Zagazig University Hospitals. Iran J Pediatr.

[27] Azab SF, Sherbiny HS, Saleh SH (2015). Reducing ventilator-associated pneumonia in neonatal intensive care unit using "VAP prevention Bundle": A cohort study. BMC Infect Dis.

[28] American Thoracic Society; Infectious Diseases Society of America (2005). Guidelines for the management of adults with hospital-acquired, ventilator-associated, and healthcare-associated pneumonia. Am J Respir Crit Care Med.

[29] Grgurich PE, Hudcova J, Lei Y, Sarwar A, Craven DE (2012). Management and prevention of ventilator-associated pneumonia caused by multidrug-resistant pathogens. Expert Rev Respir Med.

